# Approaches for the Treatment of SARS-CoV-2 Infection: A Pharmacologic View and Literature Review 

**DOI:** 10.22037/ijpr.2020.113821.14506

**Published:** 2020

**Authors:** Hajar Mohammadi Barzelighi, Bahram Daraei, Farzaneh Dastan

**Affiliations:** a *Biosun Pharmed Factory, Barkat Pharmaceutical Group, Tehran, Iran. *; b *Department of Toxicology and Pharmacology, School of Pharmacy, Shahid Beheshti University of Medical Sciences, Tehran, Iran. *; c *Department of Clinical Pharmacy, School of Pharmacy, Shahid Beheshti University of Medical Sciences, Tehran, Iran.*

**Keywords:** COVID-19, SARS-CoV-2, Coronavirus, Acute Respiratory Distress Syndrome (ARDS), Pandemic

## Abstract

The emergence of a novel Coronavirus disease (COVID-19) inducing acute respiratory distress syndrome (ARDS) was identified in Hubei province of China in December 2019 and rapidly spread worldwide as pandemic and became a public health concern. COVID-19 disease is caused by a new virus known as SARS-CoV-2 (Severe Acute Respiratory Syndrome Coronavirus 2), which has recently offered many challenges and efforts to identify effective drugs for its prevention and treatment. Currently, there is no proven effective approach and medication against this virus. Quickly expanding clinical trials and studies on Coronavirus disease 2019 increase our knowledge regarding SARS-CoV-2 virus and introduce several potential drugs targeting virus moiety or host cell elements. Overall, 3 stages were suggested for SARS-CoV-2 infection according to the disease severity, clinical manifestations, and treatment outcomes, including mild, moderate, and severe. This review aimed to classify and summarize several medications and potential therapies according to the disease 3 stages; however, it is worth noting that no medication and therapy has been effective so far.

## Introduction

New Coronavirus, SARS-CoV-2 (Severe Acute Respiratory Syndrome Coronavirus 2), is the causative agent of Coronavirus disease 2019 (COVID-19), which has recently emerged as a human infectious disease originated in Wuhan, Hubei province, China since December 2019. This virus has been distributing fast in China and other countries so that at present (June 10^th^), 7,145,539 infected cases and 408,025 deaths were confirmed from worldwide countries (1, 2). The case fatality rate (CFR) of this viral infection at first was reported as 15% but subsequently decreased to 4-11%; however, it should be noted that this virus is highly transmissible (3). The World Health Organization (WHO) declared SARS-CoV-2 infection as a pandemic on March 11, 2020 (1). SARS-CoV-2 is a member of Betacoronaviruses, which contains former recognized Coronaviruses such as SARS-CoV and MERS-CoV (Middle East respiratory syndrome CoV). After sequence analysis of SARS-CoV-2, it was determined that SARS-CoV-2 shares a high nucleotide sequence identity (79.5%) with SARS-CoV but is rapidly transmitted from human-to-human compared to SARS-CoV (4). This virus is an enveloped, positive-sense, and single-stranded RNA virus with approximately 30000 bp length, which contains structural proteins including spike (S), nucleocapsid (N), matrix (M), and envelope (E) as well as non-structural proteins including RdRp (nsp12) and proteases (nsp3 and nsp5) (5). 

Since the pathogenesis details of SARS-CoV-2are unclear, there is no effective and definitive treatment and vaccine against it. Thus, therapeutic guidelines against SARS-CoV-2 are mainly based on their effectiveness in earlier groups of human Coronaviruses, such as SARS-CoV and MERS-CoV. In this regard, several drugs, such as Ribavirin, Interferon, Lopinavir-Ritonavir, Corticosteroids, and Chloroquine have been used against SARS and MERS viruses for patients' treatment (6). The approaches against Coronavirus could be divided into two categories: those targeting viral elements (structural and non-structural proteins or RNA), and those targeting human cells/signal pathways and immune systems (7, 8). Overall, 3 stages were proposed for SARS-CoV-2 infection according to the illness severity, clinical manifestations, and clinical and treatment outcomes, including mile, moderate, and severe (9). The spectrum of clinical symptoms of SARS-CoV-2 infection includes: mild type and usually asymptomatic infection involving only the upper respiratory tract, common or moderate type with pulmonary infiltration, and severe type resulting in respiratory distress and severe need to intensive care or intubation (10). At this moment, several medications are being used in clinical trials based on *in-vitro* activities and limited clinical experience against SARS-CoV-2(11). This review was mainly focused on and reviewed new potential therapies of SARS-CoV-2 infection based on the illness 3 stages; therefore, literature and clinical trials published from January to April 2020 were reviewed, especially those about medications, included in the treatment guidelines and those compounds, proposed against SARS-CoV-2 infection. 

**Medications for Stage I (mild) or Early Infection**

Treatment at Stage I is principally targeted to symptomatic relief in order to prevent the progression and severity of the disease using antiviral therapies (9).

*Chloroquine and Hydroxychloroquine*

**Figure 1:**
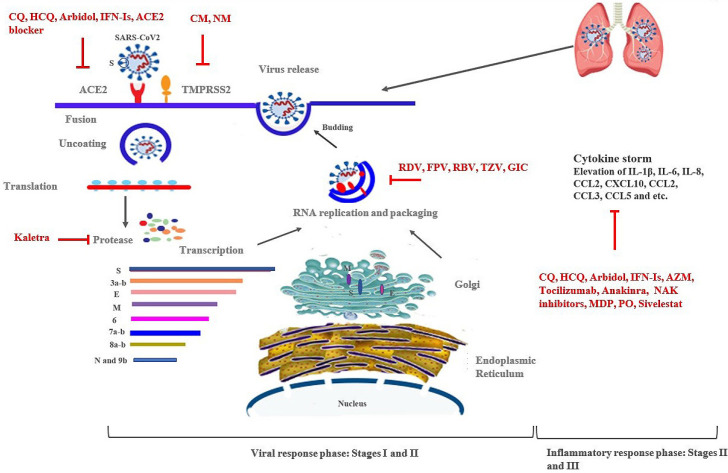
Schematic representation of approaches for the treatment of SARS-CoV-2 infection

Chloroquine (CQ) as well as Hydroxychloroquine (HCQ) are both 4-aminoquinoline drugs which are used as the drug of choice for the prevention and treatment of malaria and amoebiasis infections, and as anti-inflammatory agents for the treatment of immunological disorders such as rheumatoid arthritis, lupus erythematosus, anti-phospholipid syndrome, Sjögren’s (SHOW-grins) syndrome, and sarcoidosis (12–14). These drugs inhibit immunological progressions such as antigen presentation, expression and production of proinflammatory cytokines [tumor necrosis factor-α (TNF-α), interleukin-1β (IL-1β), and interleukin-6 (IL-6)] (13), and activation of Toll-like receptors and NLRP3 inflammasome. They also modulate the functions and differentiation of T cell subsets and induce apoptosis in peripheral blood lymphocytes (13, 15 and 16) (Figure 1) (Table 1). It was revealed that broad-spectrum antiviral activities of CQ (against H5N1 and SARS-CoV infected patients) are via elevating endosomal pH needed for virus/cell fusion, interfering with glycosylation of cellular receptors (angiotensin-converting enzyme 2, ACE2), interfering with the process of leaving viral RNA inside the cell, and thus blocking viral replication (14, 17). Also, the antiviral activities of CQ and HCQ against SARS–CoV-2 were demonstrated *in-vitro* and in small poorly controlled or uncontrolled clinical trials (17). It was determined that CQ functioned at both entry and post-entry stages of SARS-CoV-2 infection in Vero E6 cells (6). Wang *et al.* (2020) reported the high effectiveness of CQ and Remdesivir in controlling SARS-CoV-2 infection *in-vitro* (6). Another study by Gautret *et al.* reported the correlation between HCQ treatment and reduction or disappearance of SARS-CoV-2 load in patients. They also determined that the addition of Azithromycin to HCQ was significantly effective in eliminating the virus (18). In another studies, it was found that HCQ was more potent than CQ for *in-vitro* inhibition of SARS-CoV-2 in pneumonia patients using smaller values of EC50 (17, 19). Pourdowlat *et al.* declared that HCQ with a dose of 200 mg/day could provide a relative prophylactic effect against SARS-CoV-2 infection and could be recommended for healthcare workers during the SARS-CoV-2 pandemic (20). In clinical trials, it was determined that CQ could reduce the duration of hospitalization and recover patients with SARS-CoV-2 pneumonia; thus, the administration of CQ at a dose of 500 mg twice daily for 10 days was recommended for mild, moderate, and severe forms of infection (21). Various dosages of HCQ were recommended in clinical trials for the treatment of SARS-CoV-2 infection (22). Despite the results of different studies mentioned, Molina *et al.* (2020) in France found no evidence of antiviral activity of HCQ or clinical advantage of combination therapy with HCQ and Azithromycin in hospitalized patients with severe SARS-CoV-2 infection (23). CQ could cause cardiac arrhythmias, QT prolongation, other cardiotoxicities, and retinal injury (11). Its administration in diabetic and G6PD deficiency patients should be done with caution (11). Recently, it was reported that CQ and HCQ regimens with or without Azithromycin, not only were not useful but also could be harmful in infected patients with SARS-CoV2 (24).

The Food and Drug Administration (FDA) approved the emergency use of CQ against SARS-CoV-2 infection. The European Medicines Agency (EMA) declared that several clinical trials are under investigation to generate supportive and robust data on the safety and efficacy of CQ and HCQ against SARS-CoV-2 infection (25).

*Favipiravir (Avigan®, Favilavir, T-705)*

Favipiravir (FPV) as a well-known antiviral drug has broad-spectrum antiviral activity against various viruses such as Influenza, Arenaviruses, Phleboviruses, Hantaviruses, Flaviviruses, Enteroviruses, Western equine encephalitis virus, Respiratory syncytial virus, and Noroviruses (6, 23 and 26). FPV selectively and effectively inhibits RNA-dependent RNA polymerase (RdRp) in RNA viruses (Figure 1) (Table 1). It is converted into an active form (favipiravir-ribofuranosyl-5′triphosphate: RTP) in cells and is recognized as a substrate by viral RNA polymerase, thus inhibiting RNA polymerase activity. FPV antiviral activity is reduced by the presence of purine analogs (27). FPV has been approved by Japan and China as an anti-flu drug and entered in Phase III clinical trials against SARS-CoV-2 infection. It is a therapy currently used in clinical trials against Influenza and Respiratory syncytial viruses (RSV) in USA (28).

A randomized clinical trial was performed by Chen *et al.* in which the effectiveness of FPV and Arbidol in the treatment of SARS-CoV-2 infection was compared (29). FPV was determined to accelerate the improvement of clinical manifestations such as fever, cough, and dyspnea (breathing difficulties) compared with Arbidol; however, its effectiveness in improving clinical recovery rate within 7 days was not significant compared with Arbidol. On the contrary, it was effective in improving clinical recovery rate of patients with moderate SARS-CoV-2 infection (29). In another study, the effect of FPV versus Lopinavir (LPV)/ritonavir (RTV) plus interferon (IFN)-α against SARS-CoV-2 infection was investigated via aerosol inhalation (30). FPV showed more significant effects on tomography (CT-scan) improvement, viral clearance, and drug safety in SARS-CoV-2 infected patients (30). In another research, the effect of FPV combined with Tocilizumab in the treatment of SARS-CoV-2 disease was investigated (NCT04310228), and respiratory clearance of virus, CT-scan, fever, and clinical manifestations improvement were analyzed (30). A randomized, double-blind, placebo-controlled clinical trial with three groups including FPV plus CQ, FVP, and placebo (control group) recipients is under consideration (NCT04319900); in this study, improvement of respiratory symptoms, shedding of viral nucleic acid, progression of illness to severe form, duration of fever, concentration of C-reactive protein, and improvement of lung CT are measured (NCT04319900). According to the excellent reports of China about the effect of FVP on the treatment of SARS-CoV-2 infection, several clinical trials and efforts have been carried out by many countries such as Iran, Italy, Russia, South Korea, Japan, Indonesia, and US, so far (NCT04336904) (31). 

The recommended dosage for FVP is 1600 mg twice daily for 1 day, then 600 mg twice daily for 7–10 days (29). The adverse effects of FVP includes hematopoietic tissues disorders such as diminished production of red blood cell (RBC); toxicity of testis; and increase in aspartate aminotransferase (AST), alkaline phosphatase (ALP), alanine aminotransferase (ALT), and total bilirubin (32). Its administration in pregnant women should be limited (Table 1) (32).

*Umifenovir (Arbidol®)*

Arbidol is a Russian-produced, synthetic agent with broad spectrum antiviral activity against numerous enveloped and non-enveloped viruses (33). The drug is well-known in Russia and China and is currently administered clinically in several countries but not in North America; however, it has not been approved by FDA and EMA (33, 34). The molecular mechanisms of Arbidol function include: i) inhibiting the fusion of virus with the cell membrane, ii) acting as an immunomodulatory , and iii) act as interferon-inducing agent (35) (Figure 1) (Table 1). The inhibitory effect of Arbidol has been reported against Influenza A and B, avian influenza, SARS-CoV (33), Hepatitis C (36), Hepatitis B (34), Ebola virus, Arenavirus, Human Herpesvirus 8 (HHV-8), Poliovirus, and Lassa virus (34, 37). This antiviral agent which block virus entry is notably owing to the arrest of the first step of the viral infection cycle (37). A preliminary *in-vitro* study determined the effectiveness of Arbidol in inhibiting SARS-CoV-2 infection at 10-30 μM (38). In a study by Li *et al.*,it was found that Arbidol accelerated and increased viral clearance, improved lung CT image, and reduced the necessity for high flow nasal catheter oxygen therapy (39). In another experimental study, Arbidol was announced as an option for the treatment of SARS-CoV-2 infection (40). In the latest guidelines of Chinese National Health Commission (NHC), Arbidol was recommended for the prevention and treatment of SARS-CoV-2 infected patients as oral administration at a dose of 200 mg every 8 h (3 times/day) for 10 days in adults (38). In the computational docking analysis to discover potential drugs, it was shown that Arbidol may target Nsp7-Nsp8 complex, Nsp14, Nsp15, E-channel, or spike protein of SARS-CoV-2 (8). In a retrospective cohort study, the efficacy of monotherapy with LPV/RTV was compared to combination therapy with LPV/RTV plus Arbidol against SARS-CoV-2 infection , showing favorable results in combination therapy (41). A randomized, open-label, multi-center study is carrying out in China on the effectiveness and safety of Arbidol hydrochloride tablets in SARS-CoV-2 pneumonia (NCT04260594). 

Arbidol has antioxidant properties and is categorized as the least toxic drug; however, it may be accompanied by significant adverse effects observed in the treatment of influenza patients, including diarrhea, nausea, dizziness, and increase in serum transaminase (Table 1) (42).

*Ribavirin (RBV) or Tribavirin*

RBV is a nucleoside analogue competing with nucleotide active site of RNA-dependent RNA Polymerase, which has broad-spectrum antiviral effects against chronic and acute HCV infection (in combination with other antiviral agents); HEV infection; RSV infection; and viral hemorrhagic fever (VHF) agents such as Lassavirus, Arenavirus, Hantavirus, Crimean-Congo hemorrhagic fever, SARS-CoV, and MERS-CoV (Figure 1) (Table 1) (43, 44). It has been approved by FDA and EMA against HCV, RSV, and hemorrhagic fever (43). In a preclinical study, the activity of RBV against SARS-CoV-2 was determined (6). 

The use of RBV in combination with other antiviral agents, such as LPV-RTV, against SARS-CoV infection was shown to reduce the risk of acute respiratory distress syndrome (ARDS) and death (44). The computational analysis and molecular docking proposed Sofosbuvir, RBV, and RDV as antiviral agents against SARS-CoV-2 (45). A clinical trial evaluated and compared the effect of combination therapy with LPN-RTV, RBV, and INF beta-1B and monotherapy with LPN-RTV on the recovery rate, viral clearance, hospitalization duration, and death in SARS-CoV-2 infected patients (NCT04276688). In another clinical trial, the safety and efficacy of RBV plus INF-alpha, LPN-RTV plus INF-alpha, and RBV- LPN-RTV plus INF-alpha were investigated in mild and moderate forms of SARS-CoV-2 infection (ChiCTR2000029387). RBV was included in Chinese Plan Edition 5 for the treatment of pneumonia (46). RBV was proposed to be administered intravenously at a dose of 500 mg every 12 or 8 h for adults (2 to 3 times/day) in combination with IFN-alpha or LPV/RTV (38). 

Several side effects have been reported for RBV, including headache, nausea, muscle pain, fever, tiredness, liver problem, red blood cell destruction, allergic reaction, and harmful effect during pregnancy on fetus (Table 1) (47). 

*Lopinavir and Ritonavir (LPV/RTV; Kaletra®)*

 These drugs were classified and approved by FDA and EMA as antiretroviral drugs, and their effects on the improvement of SARS-CoV and MRS-CoV infections were determined *in-vitro* and clinical symptoms (44, 48). LPV is a proteinase inhibitor whose serum concentrations inhibit the replication cycle of SARS-CoV and block MERS-CoV (49). RTV inhibits the metabolism of LPV by CYP3A (as potent inhibitor of CYP3A) and increases its serum concentration (49).

The large part (two-third) of the Coronavirus genome is translated to a polypeptide that encodes essential proteins involved in the viral replication and gene expression, among which two proteases, including Papain-like (PL^pro^) and 3-chymotrypsin-like (3CL^pro^), are critical for virus replication. 3CL^pro^ (3CL^pro, ^aka main protease and M ^pro^) is an appropriate target for drug therapy (50). 3CL^pro ^which is highly conserved in terms of sequence with a three-dimensional structure and approximately 306 amino acids in length, is a crucial enzyme for Coronavirus replication (50, 51). It seems that M ^protease^ is an attractive target for the treatment of SARS-CoV-2 infection due to its properties (51). Kaletra was suggested as a potential inhibitor for M ^protease^ of SARS-CoV-2 with clinically approved medicines (Figure 1) (Table 1) (52). LPV has been suggested as a potent drug against Coronaviruses according to the docking and molecular dynamic experiments (53).

Kaletra efficacy in decreasing SARS-CoV-2 load and improving clinical manifestations was shown in South Korean patients (54). In some trials, the therapeutic role of Kaletra against SARS-CoV-2 has been compared with that of Arbidol hydrochloride (NCT04255017, NCT04252885) and Traditional Chinese Medicines (TCMs) (NCT04251871). Nevertheless, no benefit was reported in the treatment of SARS-CoV-2 hospitalized adult patients with Kaletra (55). In a study performed in Singapore, the treatment of SARS-CoV-2 infected patients with Kaletra resulted in variable clinical outcomes; fever resolved and the need for supplemental oxygen decreased in 3 out of 5 patients (60%) during 3 days. In 2 out of 5 (40%) patients, nasopharyngeal swabs qRT-PCR was negative, and viral shedding was cleared. The drug adverse effects such as nausea, vomiting, diarrhea, and abnormal liver function occurred in 4 out of 5 patients (80%) (56). 

The recommended dosage is oral administration of LPV 400 mg-RTV 100 mg two times per day for 10 days, which could be administered with or without interferon (57). The other side effects include cardiac arrhythmias and hepatic failure (Table 1) (11).

*Type 1 Interferons (IFN-Is*)

IFN-Is, as a group of cytokines, including alpha and beta subtypes, are the first cytokines produced by various cell types particularly plasmacytoid dendritic cells, diagnosing viral components by pattern recognition receptors (PRR) (58). They are recognized by IFNA-R, and their interaction induces phosphorylation of transcription factor STAT1 and stimulates interferon stimulated genes (ISG) which are involved in inflammation, cell signaling, and immunomodulation process. These genes induce different functions such as deceleration of cell metabolism, induction of cytokine secretion to activate the adaptive immunity, and decrease in plasma membrane fluidity for inhibition of viral fusion or protrude (59). IFN-Is as immunomodulatory agents are used for the treatment and control of some cancers and multiple sclerosis (60, 61). 

IFN-I plays a major role in antiviral immunity and therapy (62). Several studies have been done *in-vitro* and *in-vivo* to investigate the therapeutic role of INF-I against SARS-CoV and MERS-CoV infections as monotherapy or combination therapy with LPV-RTV, RBV, and RDV. The results were different and relatively successful *in vitro* assays and in some animal models; however, there was no significant improvement in human , which may be related to the mechanism of action of SARS-CoV and MERS-CoV against IFN-I signaling pathway, limited number of patients and animals in the studies, difficulty in interpreting results in combination therapy, difficulty in determining IFN-I role, and the use of different subtypes of IFN-I in different studies (62). IFNβ1 seems to be a significant IFN-I in the treatment of Coronavirus infections due to its protective activity in lung through upregulating cluster differentiation 73 (CD73) in lung endothelial cells, inducing secretion of adenosine as an anti-inflammatory agent, and inducing discretion of vascular leakage in acute respiratory distress syndrome (ARDS) (62, 63). The administration timing of IFNβ1 plays a crucial role in the treatment, and it was proposed to be administered shortly after the infection onset (64). 

It was determined that Orf3 and Orf6 of SARS-CoV and MERS-CoV disrupted IFN-I signaling pathways (65), but these proteins in SARS-CoV-2 are truncated (66), and this virus may be more sensitive to IFN-I than other Coronaviruses (62). The significant sensitivity of SARS-Cov-2 to IFNα was observed in a study by Lokugamage *et al.* (66). Shen *et al.* found that spray formulated IFNα2b could reduce the rate of SARS-CoV-2 infection in children (67). They showed the prophylaxis role of INF-I against SARS-CoV-2 (67). In the pathophysiology of SARS-CoV-2, it was suggested that this virus stimulates excessive IFN-I antiviral responses, leading to pulmonary lesions and damages; thus, according to this hypothesis, it seems that IFN-I therapy should be limited to the early step of infection since increase in mortality rates associated with inflammatory biomarkers was observed (68). 

The Chinese therapy guidelines included IFNα administration as inhalation of 5 million U twice daily in combination therapy with RBV (38). It was suggested that combination therapy of IFN-I with LPV-RTV, RBV, or RDV may improve its efficacy; it is under investigation in clinical trials (ChiCTR2000029387). Very newly, the prospective non-controlled trial was done on 20 patients which received IFN-β-1α (44 μg) subcutaneously with conventional therapy including HCQ and LPV/RTV up 10 days (69). The outcome of study include: virus clearance, symptom decrease, and improvement in lung imaging, so the study supports the role of IFN-β-1α in the management of SARS-CoV-2 infection (69).

Totally, IFNβ1 may be considered as a safe and easy treatment against SARS-CoV-2 infection in the early stages (62). The adverse effects of IFN include flu-like symptoms, such as fever, feeling ill, headache, fatigue, muscle pain, dizziness, convulsion, hair thinning, and depression (Table 1) (70).

**Supporting agents for the treatment in Stage I**

*Azithromycin (AZM)*

AZM as an antibacterial agent with immunomodulatory and anti-inflammatory properties has some antiviral activities *in-vitro* assay against Influenza H1N1 (71), Rhinovirus (72–74), and Zika virus (75). 

There is little evidence about its effect against Coronaviruses such as SARS-CoV and MERS-CoV. AZM may reduce inflammatory responses,excessive production of cytokines, neutrophils (PMNs) chemotaxis, mucus hypersecretion and production of reactive oxygen species (ROS), as well as, increase neutrophil apoptosis, and block the activation of nuclear transcription factors, but its mechanism in viral clearance has not been detected yet (Table 1) (Figure 1) (11, 76). The adjunctive effect of AZM along with its antibacterial and immunomodulatory characteristics were observed in hospitalized adults with Influenza (77). In a study done by Arabi *et al.*, the association of AZM with mortality rate and viral RNA clearance in MERS-CoV critically ill patients was investigated during 90 days. The results indicated that AZM therapy did not reduce mortality rate and viral load (130). AZM has been used in the treatment of certain respiratory diseases such as bronchitis, cystic fibrosis, and chronic obstructive pulmonary disease (COPD) (72, 73). Since the advent of SARS-CoV-2, AZM has been used in China as an antibacterial agent to manage bacterial superinfection (78). 

AZM was used in combination with HCQ in confirmed SARS-CoV-2 infected patients with more than 12 years old at a dosage of 500 mg for 1 day, followed by 250 mg daily for 2-5 days in a study in France (18). In a study, it was found that the addition of AZM to HCQ significantly removed virus (18). Unlikely, in another study in France, no antiviral efficacy or clinical improvement was observed using HCQ or a combination of HCQ+AZM in patients with severe SARS-CoV-2 infection (23). The efficacy of HCQ in combination with AZM in 440 individuals with SARS-CoV-2 pneumonia is under investigation in a clinical trial in Brazil (79). Totally, AZM administration against SARS-CoV-2 infection is currently controversial and needs to be further investigated. Diarrhea, loose stools, nausea, cardiac arrhythmias (QT prolongation), and substantial drug-drug interactions were reported as the adverse effects of AZM ( Table 1) (11, 80). 

**Medications for Stage II (Moderate): Pulmonary Involvement, and Stage III (Severe)**

*Systemic Hyperinflammation*

Stage II of the disease is subdivided into two stages: Stage IIa (without hypoxia) and Stage IIb (with hypoxia: defined as a PaO_2_/FiO_2_ ratio <300 mmHg). The treatment at this stage consists of supportive procedures and available anti-viral therapies (9). In the Stage III, the inflammatory biomarkers are significantly increased, including IL-2, IL-6, IL-7, granulocyte-colony stimulating factor (GCSF), macrophage inflammatory protein 1-α (MIF-1 α), tumor necrosis factor-α (TNF α), C-reactive protein (CRP), ferritin, D-dimer, Troponin, and N-terminal pro B-type natriuretic peptide (NT-proBNP). On the contrary, the number of helper, suppressor, and regulatory T cells decreases. The clinical symptoms in this stage include shock, respiratory failure, vasoplegia, and cardiopulmonary collapse in some cases (9).

*Remdesivir (GS-5734; RDV)*

RDV is a mono phosphoramidate prodrug, an adenosine analog which functions after the virus enters the host cell and causes premature termination of viral RNA replication by incorporation into the structure of nascent RNA (81) (Figure 1) (Table 1); thus, it could be considered as a promising broad-spectrum antiviral drug against a wide range of RNA virus families including Coronaviridae (such as SARS-CoV , MERS-CoV, human CoVs OC43, and HCoV-229E) (82, 83), Filoviridae (such as Ebola virus: under development in clinical trials) (81, 84), and Paramyxoviridae (such as Nipah virus, Respiratory syncytial virus, and Hendra virus) (84). Numerous studies have investigated the effect of RDV on Coronaviruses both *in-vitro* (in cell cultures) and *in-vivo* (in mouse and primate animal models) (82, 83, 85 and 86). RDV efficacy in the prophylaxis and treatment of SARS-CoV and MERS-CoV infections was proven in a mouse model (85) and in a rhesus macaque model (86), respectively. The viral resistance to RDV via mutation has not yet been identified (85). According to the previous studies results, it was suggested that RDV may be effective against SARS-CoV-2 infection. Wang *et* *al.* found that EC90 value of RDV against SARS-CoV-2 in Vero E6 cells was 1.76 μM, and its working concentration is likely to be achieved against Ebola virus in non-human primate model. In their study, the efficient inhibitory effect of RDV against SARS-CoV-2 infection in human liver cancer cells (Huh-7) was also determined (6). They concluded that RDV and CQ are highly effective against SARS-CoV-2 infection *in-vitro *(6). Antiviral activity of RDV against SARS-CoV-2 in Vero6 cells with EC_50_ value of 23.15 μM was reported by Choy *et al.*; it was suggested that the combination therapy with RDV and emetine against SARS-CoV-2 infection may help reduce EC to 6.25 μM (70). RDV improved the clinical manifestations in the first case of SARS-CoV-2 infection in US (87). RDV has been administered for several hundred individuals with confirmed severe COVID-19 through compassionate use so far (88); for example, RDV was administered in 53 patients over a 10-day course through intravenous administration of 200 mg for one day, followed by 100 mg for 9 days (22, 22, and 9 participates from US, Europe or Canada, and Japan, respectively) (88). The clinical manifestations improvement was observed in 68% of patients. The adverse effects such as elevation of hepatic enzymes, diarrhea, renal impairment, rash, and hypotension were observed in 60% of patients, especially in the patients receiving invasive ventilation (Table 1) (88). 

Since COVID-19 began to spread worldwide, several trials have initiated in China and other countries to discover an effective drug. Gilead Sciences is running two Phase III clinical trials to investigate the safety and antiviral activity of RDV in individuals with moderate to severe SARS-CoV-2 disease and to compare it with standard treatment (NCT04292730 and NCT04292899). An adaptive, randomized, double-blind, placebo-controlled, Phase II clinical trial is carrying out in approximately 75 sites globally to evaluate the safety and efficacy of RDV in hospitalized patients with SARS-CoV-2 infection (NCT04280705). The recommended dosage for RDV by Gilead Sciences is 200 mg IV on Day 1, then 100 mg IV on Days 2-5 or 100 mg IV daily on Days 2-10 (NCT04292899). There is no FDA and EMA approval for RDV; however, it was recommended by EMA for SARS-CoV-2 infection in undergoing Phase III clinical trials in US and emergency use (89). 

*Tocilizumab (Actemra®)*

 It was determined that IL-6 levels are significantly increased in severe SARS-CoV-2 infection, and they seem to play a crucial role in cytokine release and severity of illness (90). Tocilizumab is a recombinant humanized (from mouse) monoclonal antibody which acts as an antagonist for interleukin-6 (IL-6) receptor and may possibly combat with cytokine storm symptoms in severely ill SARS-CoV-2 patients (Figure 1) (Table 1). Tocilizumab as an immunosuppressive drug was approved by FDA to treat rheumatoid arthritis (RA) and cytokine release syndrome (CRS) (91). In a clinical study, the efficacy of tocilizumab (dose of 400 mg, intravenously) was assessed in 21 severely ill patients with SARS-CoV-2 infection. Approximately, 91% of patients were discharged from hospital with clinical improvements such as recovery of respiratory function and CT opacity, changes in lymphocyte percentage and C-reactive protein level, and slowdown of fever after receiving only 1 dose (92); no adverse effect was reported in this study (92). The effectiveness of Tocilizumab on the treatment of a challenging SARS-CoV2 patient was reported by Dastan *et al. *(93). 

It was reported a challenging case of COVID-19 who was successfully treated with tocilizumab. A randomized, double-blind, placebo-controlled Phase III clinical trial is ongoing by Roche to evaluate the safety and efficacy of intravenous administration of tocilizumab beside standard care in hospitalized patients with SARS-CoV-2 pneumonia in comparison to placebo therapy and standard care. Patients are followed up for 60 days, and efficacy evidence including clinical symptoms status, mortality rate, the use of mechanical ventilation and intensive care unit are evaluated (19032020_MR_Actemra covid-19 trial_EN). A randomized, multi-center clinical trial is under consideration by Peking University first hospital on 150 participants with SARS-CoV-2 infection, who were divided into three groups, including FVP, tocilizumab, and FVP+tocilizumab recipients. The outcomes are evaluated based on clinical improvement rate, duration of fever, improvement of lung image, mortality rate, the rate of invasive or non-invasive mechanical ventilation use, and hospitalization time (NCT04310228). The clinical efficacy of another IL-6 receptor antagonist, known as Sarilumab, is under investigation in a randomized Phase II/III clinical trial on 300 participants with SARS-CoV-2 infection (NCT04327388). 

The adverse effects of Tocilizumab include gastrointestinal perforation (GP), infusion-related reactions, and hepatotoxicity. The administration of Tocilizumab in patients with thrombocytopenia and neutropenia should be done with caution (Table 1) (11). 

*Anakinra (recombinant human interleukin-1 receptor antagonist/Kineret)*

 Anakinra is a recombinant antagonist of human Interleukin 1 receptor, which is administered to treat rheumatoid arthritis. It may potentially prevent cytokine release storm (CRS) in the severe stage of SARS-CoV-2 infection (Figure 1) (Table 1) (22, 94). The main benefits of Anakinra regarding improved survival rate of patients with hyperinflammation sepsis shock is shown in Figure 1 (95). Currently, there is no published evidence about the safety and efficacy of Anakinra against SARS-CoV-2 infection (22); but a randomized, open-label, parallel, multicenter, 3-arm, Phase II/III clinical trial is ongoing to investigate the efficacy and safety of intravenous administration of Emapalumab, Anti-IFNγ monoclonal antibody, and Anakinra, versus standard care, in reducing and improving hyper inflammation and respiratory syndrome in SARS-CoV-2 infected patients, respectively (NCT04324021). In another prospective, randomized, interventional clinical trial, the safety and efficacy of IL-6 and IL-1 blockage would be compared to standard care in short- and long-term outcomes, and oxygenation status would be evaluated in the patients in the severe phase of SARS-CoV-2 infection (NCT04330638). The adverse effects of Anakinra include flu-like symptoms (22). 

*Numb-associated kinase (NAK) family- AAK1 and GAK -inhibitor: Ruxolitinib*

*Ruxolitinib (Jakafi and Jakavi)*

Ruxolitinib approved by FDA and EMA is a small-molecule Janus kinase (JAK1 and JAK2) inhibitor which is administered for myelofibrosis (96). Ruxolitinib acts as an immunomodulatory agent decreasing cytotoxic T lymphocytes and increasing T regulatory cells (96). The effects of JAK kinases, such as AAK1 and GAK, on cell trafficking and cell entry by viruses (Hepatitis C virus, Dengue virus, Ebola virus, and Respiratory syncytial virus) have been determined (Figure 1) (Table 1) (97). There is no evidence about the effect of JAK inhibitor on SARS-CoV-2 infection, but several clinical trials are ongoing. In an interventional study, the effect of Ruxolitinib on the treatment of SARS-CoV-2 infected patients with respiratory distress (20 participants) is under investigation, and the CT scan is evaluated. The drug is administered at a dosage of 5 mg twice daily (NCT04334044). Another interventional, single-center, non-randomized Phase II clinical trial on the treatment of SARS-CoV-2 infected patients (200 individuals) with defined hyperinflammation is ongoing in Germany (NCT04338958). A Phase III clinical trial has been initiated by Ruxolitinib administration as a treatment for SARS-CoV-2 infection in patients with cytokine storm (98). 

The adverse effects of Ruxolitinib include decreased number of red blood cells, white blood cells, and platelets; unusual bruising or bleeding; tiredness; fever; shortness of breath; certain types of non-melanoma skin cancers in some people; and change in cholesterol level. The individuals may also be at risk of developing serious infection during the treatment with Roxolitinib. It has not been administered in pregnant women or those planning to become pregnant (Table 1) (98).

*Corticosteroids*

Corticosteroids have been administered in patients with SARS-CoV, MERS-CoV, Influenza, RSV, and recently SARS-CoV-2 infections to diminish the inflammatory responses and inhibit the illness progression to severe, as well as in patients with acute respiratory distress syndrome (ARDS). Although the side effects, such as delayed viral clearance from the respiratory tract and blood due to the immunity suppression and risk of super infection, may cause the mortality rate to be increase, the complications include psychosis, diabetes, and avascular necrosis; thus, no clinical improvement may occur (91). Russell *et al.* in their study concluded that Corticosteroids should not be administered for the treatment of SARS-CoV-2 infection resulting in lung injury or shock and claimed that Corticosteroids are probably useful in the treatment of bacterial infections rather than viral infections (99). In a study conducted by Lei *et al.* in 2 hospitals of China, the efficacy of Corticosteroid (40 mg methylprednisolone once or twice per day) in the treatment of 31 SARS-CoV-2 infected patients was assessed; in this study, virus clearance time, hospitalization duration, and recovery rate of clinical symptoms with or without Corticosteroid treatment were measured (100). No association was detected between therapy and variables, and the virus clearance time in patients with Hepatitis B was longer (100). Therefore, no significance relationship was found between Corticosteroid treatment and SARS-CoV-2 infected patients’ recovery rate (100). 

In the Iranian protocol for treatment of SARS-CoV-2, the regimen of Steroid was recommended due to the rapid progression of ARDS in patients to inhibit or decrease the inflammation process (101). Generally, the administration of Corticosteroid seems to be controversial. 

*Ciclesonide (CIC)/Alvesco*

Ciclesonide (CIC)/Alvesco is a FDA approved inhaled glucocorticoid which is administered to treat asthma and allergic rhinitis (148). In an *in-vitro* study by Sangeun *et al.* (2020), antiviral activity of CIC against SARS-CoV-2 infection (IC50 = 4.33 µM) was detected (102). It has been determined that systemic administration of CIC in SARS-CoV and MERS-CoV infections should be limited due to the suppression of innate immune system leading to increased viral replication and mortality rate (103, 104). In another study by Matsuyama *et al. *, it was determined that inhalation of GIC suppressed human Coronaviruses replication, such as HCoV-229E and SARS-CoV, and it was suggested that CIC probably interacted with NSP15 intracellularly, and thus intervened with the replication of positive sense RNA virus; therefore, in this study, intracellular interaction of CIC with the replication site of positive-strand RNA virus was suggested, and EC90 value of 6.3 μM was reported for CIC against SARS-CoV-2 infection (Figure 1) (Table 1) (105). An open-labelled, randomized Phase II clinical trial on the monotherapy with CIC or combination therapy with HCQ-placebo is ongoing for the treatment of 141 patients with mild SARS-CoV-2 infection in Korea University Guro Hospital. The administered dosage includes 320µg oral inhalation of CIC twice daily for 14 days (NCT04330586). 

The adverse effects of CIC include: body and muscles pain, cough, chills, difficulty in breathing and shortness of breath, fever, headache, ear and nasal congestion, weakness and tiredness, sore and white patches in the mouth (102). 

*Camostat Mesilate (CM, NI-03) and Nafamostat Mesylate (NM, Fusan)*

 CM is an approved drug in Japan for clinical treatment of pancreatitis , it is also the inhibitor of cellular serine protease/Transmembrane Serine Protease 2 (*TMPRSS2*) which is involved in the priming of SARS-CoV-2 S protein and virus entry to lung cells (Figure 1) (Table 1) (106). The inhibitory effects of CM against the first step of SARS-CoV and MERS-CoV infections have been shown (91). SARS-CoV-2 was found to use TMPRSS2 and cellular cysteine protease CatB/L for priming, following the attachment to angiotensin-converting enzyme 2 (ACE2); therefore, another approach to prevent virus entry is the use of E-64d as a cysteine protease inhibitor (106). The impact of CM on the treatment of 180 patients with SARS-CoV-2 infection is under investigation in a randomized, parallel assignment, placebo-controlled clinical trial *by Aarhus *University in Denmark; in this trial, 2 tablets are administered every 8 h daily for 5 days, and the outcomes are compared to placebo group, and variables including safety, clinical status, mortality rate, admission in ICU, mechanical ventilation use, duration of supplemental oxygen use, and days spent for self-reported recovery are measured (NCT04321096). The safety and efficacy of combination therapy with HCQ-Camostat (Camostat 400 mg three times daily for 7 days plus HCQ 400 mg twice on Day 1, then 200 mg twice on Days 2-7) are under investigation and compared to HCQ-placebo therapy in 334 patients with moderate SARS-CoV2 infection by Heinrich-Heine University Duesseldorf (NCT04338906). The time of clinical improvement, fever slowdown, first negative SARS-CoV-2 test in nasopharynx swab and lower respiratory tract specimens as well as duration of oxygen therapy, mechanical ventilation use, hospitalization, and mortality rate are assessed in this trial (NCT04338906). 

NM is another serine protease inhibitor inhibiting potentially membrane fusion via S protein in MERS-CoV (107). 

TMPRSS2 proteolytically primes and activates a variety of proteins in viral envelope, and its crucial role in entry of Influenza viruses and Coronaviruses has been determined (107). Therefore, NM seems to be effective against SARS-CoV-2 infection. NM is administered intravenously at a concentration less than one-tenth of that required for Camostat (108). 

*ACE2 blockers*

There are several targets to block the ACE family for combating Coronavirus infections: i) Receptor Binding Domain (RBD) of S protein with 193 amino acids in length, ii) administration of ACE2 antibody preventing SARS-CoV-2 infection, iii) soluble ACE2 receptor which could bind to the S protein of SARS-CoV-2 and neutralize the virus, and IV) immunoadhesin format of ACE2, in which ACE2 is fused to Fc domain, or extracellular domain of ACE2 is fused to human IgG1 domain (ACE2-NN-Ig) (109). The effects of mentioned approaches against Coronavirus infections have been demonstrated (109). The administration of recombinant ACE2 (rACE2) was determined to reduce the level of Angiotensin II and improve acute lung injury syndrome (110). The effects of rACE2 on reducing ARDS caused by respiratory syncytial virus have also been determined (111). Recently, it has been demonstrated that human recombinant soluble ACE2 (hrsACE2)/APN01 with clinical grade could inhibit SARS-CoV-2 infection in the early stage of infection by reducing the virus recovery from Vero cells and virus infection in engineered human vessel and kidney organoids (112). A randomized, double-blind, Phase II clinical trial on the use of APN01 with placebo in 200 participants is ongoing in metacenters of Austria, Denmark, and Germany by ClinicalTrials.gov identifier (NCT00886353) (Table 1). 

*Convalescent Plasma and Immunoglobin therapy*

 Intravenous* Immunoglobulin (*IVIg) is a collection of IgG collected from thousands of healthy volunteer or individual exposed to infection diseases, microorganisms, and vaccines in endemic areas of infection diseases (113). It has been administered to treat autoimmune diseases and chronic inflammatory disorders as an antimicrobial agent against bacteria, viruses, and fungal microorganisms (113). It exerts its effects via neutralizing infectious agents or their toxins; suppressing inﬂammatory cytokine IL-6 and elevating anti-inﬂammatory cytokine IL-10 in the gut; reducing TLR-4 expression leading to an inflammation response; suppressing Fc receptors (FcRs), adhesion molecules of leukocyte, pathogenic Th1 and Th17 subsets, neutralizing pathogenic autoantibodies, as well as expanding regulatory T-cells (113). It was determined that sera of healthy adults contain antibodies against Coronaviruses and could inhibit HCoV-NL63 infection (114). It was also found that IVIg containing high-titer antibodies against RSV is beneficial in improving RSV infection in immunocompromised patients and reducing lung injury (113). It was proposed that immunotherapy with IgG could be performed to neutralize SARS-CoV-2, and that a combination of immunotherapy and antiviral drugs could provide alternative approaches against COVID-19 (113). 

The convalescent plasma therapy (CP) was recommended as an experimental treatment during the outbreaks of several viruses, such as Ebola, MERS-CoV, SARS-CoV, and avian Influenza H5N1 and H1N1 (115). CP therapy of these infections was associated with viral load, cytokine response in serum, and mortality rate (115). Plasma must be collected from recovered people suitable to donate blood. In eligible people, some criteria must be investigated: having a previous SARS-CoV-2 positive assay confirmed by a laboratory test; complete improvement of clinical symptoms at least 14 days prior to donation; negative results for HLA antibodies; negative results for SARS-CoV-2, either in the nasopharyngeal swab sample or in the blood sample; and optimal titer of SARS-CoV-2 neutralizing antibody (more than 1:320) (11). In a preliminary study by Chenguang Shen *et al.*, conducted on 5 critically ill SARS-CoV-2 infected patients, plasma transfusion was performed after mechanical ventilation and administration of antiviral drugs plus methylprednisolone. CP therapy was found to improve clinical status, including: decreasing fever and viral load, increasing PaO_2_/FiO_2 _ratio and neutralizing antibody against SARS-CoV-2, and resolving ARDS (115). The guidelines for screening CP donors against SARS-CoV-2 have been determined (Table 1) (116). 

In the retrospective study, IVIG as adjuvant treatment in 58 cases of severe or critical illness of SARS-CoV-2 patinents in the intensive care unit of Wuhan Hospital could reduce the mechanical ventilation and hospital length in 48h, as well as the mortality rate (117).

Several antibodies against virus compartments and host immune system modulators have been proposed against SARS-CoV-2, including Certolizuman (anti-TNFα), BXT-25 as an anti-necrosis drug composed of oxygen careers with a copolymer stabilizer (118), Ampligen^®^ (rintatolimod) as an immune modulator, anti-C5a monoclonal antibody (IFX-1), Leronlimab (PRO 140) as a humanized IgG4 monoclonal antibody targeting the CCR5 receptor on T lymphocytes (CCR5 antagonist) (118), Camrelizumab as an anti-PD-1, and thymosin as a polypeptide hormone which has been registered to be included in a clinical trial for the treatment of SARS-CoV-2 infection by Chinese (118). Meplazumab (anti-CD147) (119), Dupilumab (anti-IL-4R), Brilacidin, as a new drug representing new class of antibiotic which has non-peptide structure and broad spectrum on superbug (187) and Nanoviricide^® ^can be examined for the effects on SARS-CoV-2 infection (118). 

Nanoviricide^®^ was developed as anti-SARS-CoV-2 in US (118) based on a broad-spectrum virus-binding ligand which models the specific human cell-surface receptor such as ACE2 and is chemically attached to a nanomicelle for virus binding. Once a virus and nanoviricide join together, the virus lipid envelope is fused in nanomicelle polymer and entrapped (118). The manufacture and production of human monoclonal antibodies against SARS-CoV-2 strains isolated from survived individuals was established by Vir Biotechnology and WuXi Biologics (118).

*Cell therapy*

 At present, there is no approved stem cell-based therapeutic approach for the prevention and treatment of SARS-CoV-2 infection. However, recently, the use of mesenchymal stem cells (MSCs) has been introduced as a new therapeutic approach against SARS-CoV-2 infection (120). In a case study reported from China, allogeneic human umbilical cord MSC (hUCMSC) was administered intravenously three times by 5 × 10^7 ^hUCMSC in the critically ill patients with ventilator, intensive therapy, and liver injury. The ventilator was switched off 4 days after the second cell infusion, and the number of T cell lymphocytes returned to normal level (120). In another study (121), the effect of single intravenous infusion of MSC (1 × 10 cells per kilogram of weight) was assessed on the clinical outcomes of 7 patients including one critically severe-ill, four severely ill, and 2 non-severely ill patients. The clinical manifestations in all patients included breath shortness, high fever, and oxygen saturation (121). The clinical symptoms improved in all patients, and the effects of cell therapy on increasing lymphocytes with shift to CD4^+^ T cells and dendritic cells and decreasing inflammatory cytokines were reported (121). 

CYNK-001 is considered as the only cryopreserved allogeneic, off-the-shelf NK cell therapy developed based on the placental Hematopoietic stem cells (HSCs) and as a potential treatment option for various hematologic and solid cancers (122). The use of CYNK-001 as a cell therapy approach for SARS-CoV-2 infection is under investigation (Table 1) (118).

**Table 1:** Summary of medications proposed for the treatment of SARS-CoV-2 infection

**Agent/Approach**	**Mechanism of Action**	**Dosage**	**Adverse Effects**	**Special Population**	**Comments**
Chloroquine (CQ)	-As an immunomodulatory agent via inhibiting immunological progressions such as antigen presentation, expression and production of proinflammatory cytokines - Interfering with virus/cell fusion via elevating endosomal pH and glycosylation of ACE2 - Blocking virus replication	500 mg twice a day orally for 10 days in mild, moderate, and severe forms of the infection	- Cardiac arrhythmias, QT prolongation - Other cardiotoxicities - Retinal injury -Anorexia -Diarrhea -Nausea	- Should be used with caution in diabetic and G6PD deficiency patients -May be used during pregnancy but has not been formally assigned to a certain pregnancy category by FDA	Efficacy and safety of CQ for the treatment or prevention of SARS-CoV-2 infection need to be more investigated
Hydroxychloroquine (HCQ)	The mechanism of action is similar to CQ	Optimal dosage and duration are not known	Similar to CQ	Similar to CQ	Efficacy and safety of HCQ for the treatment or prevention of SARS-CoV-2 infection need to be more investigated
Remdesivir (RDV)	Adenosine analog which inhibits RNA dependent RNA Polymerase (RdRp)	A 10-day period: one day 200 mg intravenously, followed by 9 days 100 mg	-Elevation of hepatic enzymes - Diarrhea - Renal impairment - Rash -Hypotension	Advised by WHO to be administered with caution and careful risk-benefit analysis before using in pregnant women	More data and investigations are needed about its safety and efficacy
Favipiravir (FPV)	Inhibiting RNA-dependent RNA polymerase (RdRp)	1600 mg twice daily for 1 day, then 600 mg twice daily for 7–10 days	- Hematopoietic tissues disorders -Toxicity of testis -Increase in aspartate aminotransferase (AST), alkaline phosphatase (ALP), alanine aminotransferase (ALT), and total bilirubin	Its administration in pregnant women should be limited, and may be classified in Category C	Additional data about its safety, efficacy, dosage, and treatment duration are needed
Arbidol	- As an immunomodulatory agent - Inhibiting virus fusion - As an interferon-inducing agent	Oral administration of 200 mg for adults, 3 times per day for 10 days	-As the least toxic drug - Diarrhea - Nausea - Dizziness -Increase in serum transaminase	It is classified in Category C in pregnancy	It is included in the treatment guidelines of some countries (81)
Ribavirin (RBV)	Nucleoside analogue inhibiting RNA dependent RNA Polymerase (RdRp)	500 mg for adults intravenously, 2 to 3 times daily in combination with IFN-alpha or LPV/RTV	-Headache -Nausea - Muscle pain - Fever - Tiredness - Liver problem - Red blood cell destruction - Allergic reaction	It is classified in Category X in pregnancy	Its efficacy and safety for the treatment of SARS-CoV-2 infection have not been definitely established and need to be more investigated
Lopinavir and Ritonavir (Kaletra)	3CL^pro^ inhibitor	LPV 400 mg-RTV 100 mg, twice daily, orally for 10 days	-Cardiac arrhythmias -Hepatic failure	It is classified in pregnancy Category C	Its efficacy for the treatment of SARS-CoV-2 infection has not been definitely established and needs to be more investigated
Type 1 Interferons (IFN-Is)	-As an immunomodulatory agent -Inflammation - Inducing cytokine secretion - Decreasing plasma membrane fluidity - Inhibiting viral fusion or budding - Activating adaptive immunity	5 million U inhalation, twice daily in combination therapy with RBV	-Fever - Feeling ill -Headache - Fatigue - Muscle pain, dizziness - Convulsion hair thinning - Depression	It is administered even in children	It is included in the treatment guidelines of some countries for SARS-Cov-2 treatment and prophylaxis in combination with LPV-RTV, RBV, or RDV
Azithromycin (AZM)	-As an immunomodulatory and anti-inflammatory agent via decreasing PMNs chemotaxis, preventing mucus hypersecretion, reducing the RO production, increasing neutrophil apoptosis, and blocking the activation of nuclear transcription factors	In individuals with more than 12 years old at a dosage of 500 mg on Day 1, then 250 mg daily until 2-5 days with HCQ	-Diarrhea - Loose stools - Nausea - Cardiac arrhythmias (QT prolongation) - Drug interactions	It is classified in pregnancy Category B	AZM administration against SARS-CoV-2 infection is currently controversial and needs to be further investigated
Tocilizumab	Antagonist of IL-6 receptor -Decreasing IL-6 and probably cytokine storm	At a dose 400 mg, intravenously	-Gastrointestinal perforation (GP) - Infusion related reactions - Hepatotoxicity	-Should be administered with caution in thrombocytopenia and neutropenia -It is classified in pregnancy Category C	Its administration is controversial
Anakinra	Antagonist of human Interleukin 1 receptor and probably preventing cytokine storm	Infusion four times daily for 15 days. 400 mg/day in total, it is divided into 4 doses given every 6 hours	-Fever -Chills -Mouth sores -Weight loss -Shortness of breath -Sore throat -Tiredness		It is under investigation in clinical trials
Ruxolitinib	-As an immunomodulatory agent via decreasing cytotoxic T lymphocytes and increasing regulatory cells -Interference with virus entry	5 mg twice daily	-Decreased cell counts (RBC, WBC and platelet), -Unusual bruising or bleeding - Tiredness - Fever - Shortness of breath - Change in cholesterol level	It is classified in pregnancy Category C	It is under investigation and needs to more evidence and data
Corticosteroids	-Probably diminishing inflammatory response	40 mg methylprednisolone once or twice per Day	- Increase in appetite, -Weight gain, - Insomnia, -Fluid retention, and -Mood changes, such as feeling irritable, or anxious	-	Its administration seems to be controversial
Ciclesonide (CIC)	Interfering with the replication of positive sense RNA virus	320 µg oral inhalation twice daily for 14 days	-Body and muscles pain - Cough - Chills - Difficulty in breathing and shortness in breath - Fever - Headache - Ear and nasal congestion - Weakness and tiredness - Sore and white patches in mouth		There is no evidence for CIC administration against SARS-CoV-2 infection
Camostat Mesilate (CM) and Nafamostat Mesylate (NM)	Blocking virus entry to cells via inhibiting cellular serine protease TMPRSS2	Camostat 400 mg three times daily, for 7 days	-	-	It is under investigation in clinical trials, and there is no evidence
ACE2 blockers	Blocking virus entry to cells	-	-	-	It is under investigation in clinical trials
Convalescent Plasma and Immunoglobin therapy	-Neutralizing infectious agents -Suppressing IL-6 - Elevating IL-10 - Reducing TLR-4 expression -Suppressing Fc receptors (FcRs), adhesion molecules of leukocyte, pathogenic Th1 and Th17 subsets -Expanding regulatory T-cells	-	-	-	It is under investigation in clinical trials
Cell therapy	Increasing lymphocytes with shift to CD4^+^ T cells and DC and decreasing inflammatory cytokines	Based on the cell type, it is different in various studies	-	-	It is under investigation in clinical trials

## Conclusion

The COVID-19 pandemic exhibits the greatest global public health concern. Extensive and expanding volume of clinical trials has been launched to investigate the potential approaches and therapies for SARS-CoV-2 infection. Whereas some medications such as CQ, HCQ, Arbidol, RBV, and so on have been entered in the therapeutic guidelines of some countries, no therapy has been recorded as definite and effective treatment for COVID-19 to date, and efforts continue. 

WHO stated that no drug therapy until now as treatment or prophylaxis is recommended outside of the context of clinical trial (123). The publications on these medicines are mostly observational, with few controlled clinical trials; and do not provide high-quality evidence in favor of any of these agents. In addition, important side effects should be considered regarding such administrations. 
